# Molecular Epidemiology of Toscana Virus in Northern and Central Italy Using Metagenomic Next-Generation Sequencing

**DOI:** 10.3390/pathogens15030338

**Published:** 2026-03-21

**Authors:** Giulia Alessandri, Giada Beligni, Gianni Gori Savellini, Alessandra Mistral De Pascali, Federica Gobbo, Fabrizio Montarsi, Davide Mileto, Letizia Rizzo, Maria Grazia Cusi

**Affiliations:** 1 Virology Unit, Department of Medical Biotechnologies, University of Siena, 53100 Siena, Italy; giulia.alessandri@student.unisi.it (G.A.); giada.beligni@unisi.it (G.B.); gianni.gori@unisi.it (G.G.S.); l.rizzo2@student.unisi.it (L.R.); 2 Department of Surgical and Neuronal Sciences, Alma Mater Studiorum, University of Bologna, 40138 Bologna, Italy; alessandra.depascal3@unibo.it; 3 Laboratorio di Entomologia Sanitaria e Patogeni Trasmessi da Vettori, Istituto Zooprofilattico Sperimentale delle Venezie, Viale del Università 10, 35020 Legnaro, Italy; fgobbo@izsvenezie.it (F.G.); fmontarsi@izsvenezie.it (F.M.); 4 SC Microbiologica Clinica, Virologia e Diagnostica Bioemergenze, ASST Fatebenefratelli Sacco, Polo Universitario “L. Sacco”, Via G.B. Grassi 74, 20157 Milano, Italy; davide.mileto@asst-fbf-sacco.it; 5 Microbiology and Virology Unit, Santa Maria delle Scotte University Hospital, Viale Bracci 1, 53100 Siena, Italy

**Keywords:** Toscana virus, mNGS, phylogenetic analysis

## Abstract

Toscana virus (TOSV) is an arthropod-borne virus, transmitted by sandflies, which represents a major cause of aseptic meningitis in Mediterranean countries during summer months. Despite its epidemiological importance, recent genomic data on circulating Italian strains remain limited. We performed comprehensive phylogenetic and genotypic characterization of 34 TOSV isolates (32 obtained from human biological samples and 2 from sandfly homogenates) collected between 2022 and 2025 from Northern/Central Italy. All the sequenced isolates clustered within Lineage A, with strains circulating in Tuscany showing significantly lower intra group genetic divergence (*p* < 0.05), indicative of compartmentalized local circulation. Both S and M segments exhibited negative selection and identified non-synonymous mutations deserving functional investigation. This study documents stable Lineage A predominance across Italian regions, with Tuscany showing distinct phylogeographic structuring. mNGS proves effective for TOSV genomic surveillance, supporting refined public health strategies, including targeted sandfly control in endemic foci.

## 1. Introduction

Toscana virus (TOSV) is an arthropod-borne virus belonging to the order Bunyavirales, family Phenuiviridae [1], primarily transmitted by sandflies of the Phlebotomus genus, particularly *Ph. perniciosus* and *Ph. Perfiliewi* [2]. First isolated in 1971 in Tuscany, Italy, TOSV has emerged as a significant cause of viral meningitis in Mediterranean countries and is currently recognized as one of the leading etiological agents of meningitis and encephalitis during the summer season [3,4]. Consequently, human infections are seasonal, with most cases diagnosed during the warm season, with a peak between June and September, corresponding to the highest vector circulation and activity period.

Although most infections are asymptomatic or associated with mild influenza-like symptoms, and therefore clinically undiagnosed, a subset of cases involves neuroinvasion, leading to severe central nervous system (CNS) manifestations such as meningitis and meningoencephalitis [5]. Despite its epidemiological relevance, the viral replication cycle within these vectors remains poorly characterized, and limited information is available regarding vector competence and virus–vector interactions that may influence transmission dynamics [6,7].

TOSV is an enveloped virus with a segmented negative strand RNA genome comprising three noncovalently closed, circular RNA fragments: the large (L) segment encodes the RNA-dependent RNA polymerase, the medium (M) segment encodes the envelope glycoproteins (Gc and Gn) and a non-structural protein (NSm), and the small (S) segment contains open reading frames for both the nucleoprotein (N) and the non-structural protein (NSs), encoded by an ambisense coding strategy, with a total genome length of approximately 12 kb [1].

Phylogenetic analyses have revealed two primary lineages, A and B, historically associated with Italy and Spain, respectively [8], with a third lineage, C, recently identified in Croatia and Greece; however, it has been characterized only by partial sequences, and whole-genome sequences or viral isolates are not yet available [9,10].

Intra-lineage relationships remain poorly investigated, and no significant differences in virulence or clinical presentation among lineages have been reported to date. Molecular epidemiology studies have documented the circulation and spread of TOSV lineages throughout the Mediterranean region over the last decades [11]. Diagnosis of TOSV infection is typically based on the detection of viral RNA in cerebrospinal fluid (CSF), the identification of specific IgM antibodies in serum during the acute phase, or evidence of seroconversion [12]. Although viral isolation is not routinely employed for diagnostic purposes, it remains essential for genetic characterization and phylogenetic analyses, which are critical for understanding viral evolution, host adaptation, and potential molecular determinants of pathogenesis. In this context, metagenomic Next-Generation Sequencing (mNGS) has recently emerged as a powerful approach for comprehensive pathogen detection and genomic characterization [13].

This new approach allows the simultaneous identification of bacteria, viruses, fungi, and parasites directly from clinical fluids such as blood, cerebrospinal fluid, or bronchoalveolar lavage [13]. Compared to traditional methods such as targeted PCR or culture, mNGS can detect co-infections, emerging pathogens, or rare microorganisms that grow poorly in vitro, and also provide relevant genetic information, such as antimicrobial resistance genes [14,15]. Thanks to these features, mNGS is becoming a valuable tool for rapid and precise diagnoses, especially in complex cases not resolved by conventional testing.

In this study, we conducted a comprehensive phylogenetic and genotypic characterization of TOSV strains detected over the past three years. Our work provides updated insights into the circulation, genetic diversity, and evolutionary dynamics of TOSV in some central–northern regions of Italy, contributing to a more detailed understanding of its epidemiology and informing public health strategies for surveillance and prevention.

## 2. Materials and Methods

### 2.1. Viral Isolates

TOSV strains were isolated in Vero E6 cells (ATCC CRL-1586) from biological samples (cerebrospinal fluid, urine, plasma, and serum) collected from patients with meningitis for whom an infectious etiology was suspected. Cells were maintained in Dulbecco’s Modified Eagle Medium (DMEM; EuroClone, Milan, Italy) supplemented with 100 U/mL penicillin–streptomycin (EuroClone, Milan, Italy) and 5% heat-inactivated fetal bovine serum (FBS; EuroClone, Milan, Italy) and incubated at 37 °C in a humidified atmosphere containing 5% CO_2_. Culture supernatants were collected upon the appearance of cytopathic effects and stored at −80 °C. The presence of the viral genome in the isolates was confirmed by TOSV-specific RT-PCR.

Viral isolates were obtained from different regions of northern and central Italy, including Tuscany (Virology Laboratory, S. Maria delle Scotte Hospital, Siena, Italy), Lombardia (Virology Laboratory, Luigi Sacco Hospital, Milan, Italy), and Emilia-Romagna (Great Romagna Hub Laboratory, Pievesestina, Forlì-Cesena, Italy). Specifically, twenty isolates were from the Bologna area (samples 3–22), ten from the Siena area (samples 23–32), and two from Milan (Lombardia) (samples 33–34). Additional viral isolates were obtained from homogenates of sandflies provided by the Istituto Zooprofilattico Sperimentale delle Venezie (IZSVe, Veneto, Italy) (samples 1,2).

### 2.2. Next-Generation Sequencing

Next-Generation Sequencing (NGS) was initially performed on two sandfly homogenates (samples 1 and 2) and two clinical samples (samples 27 and 30), for which informed consent was available, to validate the workflow and to verify that no mutations were introduced by virus isolation. Samples with insufficient original volume or lacking informed consent were sequenced after virus isolation. The nucleic acid extraction was performed by using the automatic extractor EZ1 and the EZ1^®^ DSP Virus Kit (Qiagen Gmbh, Hamburg, Germany).

Before starting the Next-Generation Sequencing (NGS) protocol, the quantity of the total nucleic acid has been assessed with a Qubit DNA high-sensitivity assay kit and Qubit 3.0 fluorometer (Thermo Fisher Scientific, Waltham, MA, USA).

Thirty-four samples (viral isolates) were selected for NGS analysis. Nucleic acid extraction and quantification was addressed as described above. Library preparation was carried out following the Illumina RNA Prep with Enrichment kits (Illumina S.r.l. Milan, Italy) according to the manufacturer’s instructions. Briefly, 8.5 μL of extracted RNA, was denatured, followed by first- and second-strand DNA synthesis. This was followed by tagmentation, which uses enrichment bead-linked transposomes (EBLT) to tagment double-stranded cDNA. This process fragments cDNA and adds adapter sequences. After tagmentation, the fragments were purified and amplified to add index adapter sequences for dual indexing. An index set, containing 96 unique, single-use Illumina DNA/RNA UD indexes, was used. Following clean-up, 7.5 μL of the library was used for hybridization using oligos from the Viral Surveillance panel 2 (https://www.illumina.com/products/by-type/sequencing-kits/library-prep-kits/viral-surveillance-panel.html accessed on 15 January 2026).

This was followed by bead-based capture of hybridized probes, amplification, clean up, and quantification of the enriched library. Normalized libraries diluted to an equimolar concentration of 10 pM were then pooled and denatured according to Illumina’s instructions and charged on a V2 micro MiSeq 300 cycles flow cell (Illumina S.r.l. Milan, Italy). FastQ files were analyzed with the BaseSpace™ platform by Illumina with the Dragen targeted microbial app (Illumina S.r.l. Milan, Italy).

### 2.3. Phylogenetic Analysis

Sequences were aligned using the Geneious software (version 2025.0.2) with the MUSCLE algorithm, using the default parameters (three iterations and gap opening penalties). After, calculation of pairwise distances was used to determine how similar or different each sequence is from the others. The phylogenetic tree was generated using the Maximum Likelihood algorithm using the Jukes Cantor distance model, which assumes equal and constant mutation rates between all nucleotide pairs. Bootstrapping and reconstitution were carried out with 1000 replicates to obtain the confidence level of the phylogenetic tree.

Sequence homology comparison was carried out with reference sequences representing the two main TOSV lineages: TOSV SI-1812 (Acc. Number EU327772.1 for segment S and OR105998.1 for segment M), TOSV SI-121283 (Acc. Number JF330274.1 for segment S and JF330280.1 for segment M), TOSV strain TI52 (Acc. Number JX867536.1 for segment S and JX867535.1 for segment M), TOSV SI-283660 (Acc. Number MN940423.1 for segment S and MN940433.1 for segment M) and TOSV strain FO_100724 (Acc. Number PV454359.1 for segment S and PV454360.1 for segment M) for Lineage A; TOSV strain H4906 (Acc. Number KU922125.1 for segment M and KU922126.1 for segment S), TOSV/Spain/LCR_853/2019 (Acc. Number OP554810.1 for segment S and OP554809.1 for segment M), TOSV/P51 (Acc. Number KU204978.1 for segment S and KU204979.1 for segment M) and TOSV strain 9028 (Acc. Number KU904263.1 for segment S and KU904264.1 for segment M) for Lineage B.

### 2.4. Evolutionary Rate and Selection Analysis

All the sequences were aligned using the MUSCLE algorithm on the Geneious software. SLAC methods (www.datamonkey.org, accessed on 20 January 2026) were used to calculate ω values (ω = dN/dS) of TOSV, where dN stands for the non-synonymous substitution rate and the dS is for the synonymous substitution rate.

## 3. Results

During the last three years, residual material of PCR-positive samples with a diagnostic request for TOSV was used to infect Vero E6 cells. Supernatants were then collected at the onset of cytopathic effect. Metagenomic sequencing and analysis followed. In addition, two isolates from sandfly homogenates, gently provided by IZSVe of Veneto, were also analyzed.

To validate the NGS approach and assess the potential impact of cell-induced virus genetic variability, we initially performed direct sequencing on two sandfly homogenates (samples 1 and 2), two clinical samples (samples 27 and 30) for which informed consent from patients has been obtained and the relative culture isolates. Resulting sequences were then compared. No additional mutations were observed between the original material and virus isolates; therefore, the remaining samples (whose original volume was insufficient for direct sequencing) were subjected to virus isolation prior to NGS analysis.

A total of thirty-four isolates from different Italian regions (Lombardia, Emilia Romagna, Tuscany and Veneto) were selected for sequencing using a metagenomic Next-Generation Sequencing (mNGS) approach.

**Figure 1:**
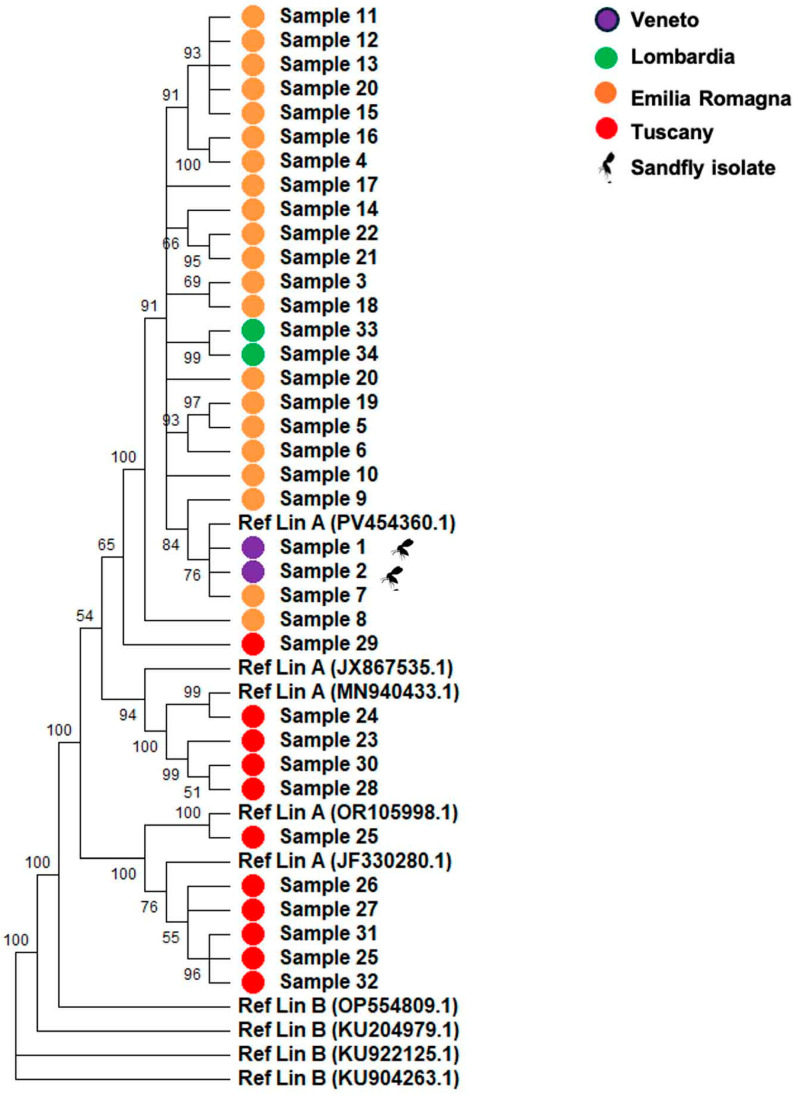
Segment S phylogenetic analysis. Samples have been sequenced and the relative consensus sequence were used to create the phylogenetic tree taking under consideration the Lineage A (EU327772.1, JF330274.1, JX867536.1, MN940423.1, PV454359.1) and B (KU922126.1, OP554810.1, KU204978.1, KU904263.1). All the samples are clustered or strictly closed to the Lineage A.

TOSV was identified in 100% of the sequenced samples with a mean coverage of 90% and an average depth of 250X. Consensus sequences were aligned using both CLUSTAL and MUSCLE algorithms, applying a minimum GC threshold of 50%. Phylogenetic analyses were conducted using reference sequences representative of Lineage A and Lineage B. Due to insufficient coverage, the L (large) segment was excluded from phylogenetic reconstruction; analyses were therefore restricted to the S (small) and M (medium) segments. As expected for an RNA virus, TOSV displayed a high degree of genomic variability, reflecting the intrinsic error-prone nature of RNA-dependent RNA polymerase replication and the continuous selective pressure acting on the viral population. Phylogenetic reconstruction of the S segment (Figure 1), performed using EU327772.1, JF330274.1, JX867536.1, MN940423.1, PV454359.1 (Lineage A) and KU922126.1, OP554810.1, KU204978.1, KU904263.1 (Lineage B) as references, showed that all samples clustered within or near Lineage A. Several well supported subclades were observed (bootstrap = 100), with short terminal branches indicating limited intra cluster diversity and suggesting a recent common ancestry. None of the samples clustered with the Lineage B reference sequence (KC776214.1), which formed a distinct and strongly supported branch (bootstrap = 90). Mean evolutionary distances were d = 0.010 (SD 0.0018) from Lineage A and d = 0.331 (SD 0.010) from Lineage B. The dN/dS ratio for the S segment was < 1 (ω = 0.02), consistent with prevailing negative selection.

**Figure 2:**
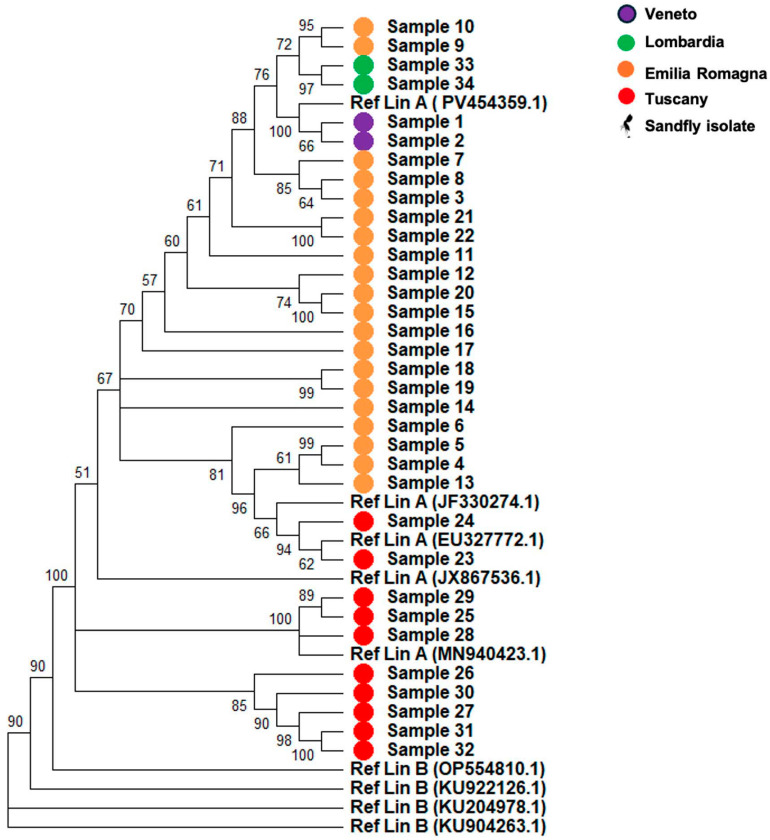
Segment M phylogenetic analysis. Samples have been sequenced and the relative consensus sequence were used to create the phylogenetic tree taking under consideration the Lineage A (OR105998.1, JF330280.1, JX867535.1, MN940433.1, PV454359.1) and B (KU922125.1, OP554809.1, KU904264.1, KU204979.1).

Similarly, analysis of the M segment (Figure 2) confirmed that all samples grouped within or in proximity to Lineage A, with no clustering observed with Lineage B. Overall branch lengths were short, indicating limited divergence, and most internal nodes showed high bootstrap support (≥91%), although a few displayed moderate support (≈51–76%). Mean evolutionary distances were d = 0.016 (SD 0.0010) relative to the Lineage A reference and d = 0.253 (SD 0.016) relative to Lineage B. The dN/dS ratio for the M segment was also <1 (ω = 0.48), further supporting the presence of negative pressures. Original high-fidelity phylogenetic trees of both S and M segments are reported in the Figures S1 and S2.

Mutation analysis identified 73 nucleotide mutations across the S segment (1869 nucleotides) and 138 across the M segment (4209 nucleotides), corresponding to a 3.91% (segment S) and 3.28% (segment M) identified variable sites (Table S1). These equate to mutation rates of 3.84 × 10^−2^ per site (S segment) and 3.27 × 10^−2^ per site (M segment). The identified polymorphisms exhibited broad distribution across both genomic segments, reflecting the error-prone nature of RNA-dependent RNA polymerase replication under continuous selective pressure.

Translation of the consensus sequences identified seven non-synonymous mutations in the S protein and nine in the M protein (Table 1).

In particular, two mutations in the NSs protein of the S segment (K→M and R→T) and two in the NSm protein of the M segment (D→S and G→R) are predicted to be structurally disruptive missense substitutions, whereas the remaining mutations are unlikely to cause substantial alterations in the proteins. Nevertheless, these changes are not expected to significantly affect viral replication, as nearly all analyzed viruses originated from strains isolated from patients with neurological disease, including the reference TOSV strain. To assess regional genetic diversity, pairwise evolutionary distances (Jukes–Cantor) were calculated for S/M segments, grouping samples by geographic origin (Table 2). Tuscan TOSV samples (20–29, Siena; *n* = 10) formed a distinct, well supported subclade (bootstrap 95%), separated from those of Emilia-Romagna (*n* = 22), Lombardia (*n* = 2), and Veneto (*n* = 2). Mean intra Tuscany evolutionary distances (Jukes–Cantor model) were significantly lower [S segment: d = 0.030 (±0.004); M segment: d = 0.039 (±0.003)] than inter regional comparisons [S: d = 0.043–0.050; M: d = 0.037–0.049; *p* < 0.05, unpaired *t*-test], including aggregated extra Tuscany variability (*n* = 24; S: d = 0.044; M: d = 0.061), with maximum divergence against Veneto sandfly isolates (*p* = 0.023). These data indicate compartmentalized local transmission in Tuscany.

## 4. Discussion

Toscana virus (TOSV), discovered in 1971, is now considered one of the leading etiological agents of meningitis and encephalitis during the summer season in the Mediterranean region [1]. Molecular epidemiology studies have widely documented the circulation and spread of TOSV lineage throughout the Mediterranean region during the last years [4,6]. To date, based on virus isolation and genetic characterization by sequencing, three distinct co-circulating lineages of TOSV have been classified (A, B and C), accordingly to their distinct geographical localization [8]. There is now indisputable evidence that TOSV is also present in North Africa (Algeria, Morocco, Tunisia), in the Kosovo and Bosnia Herzegovina regions of the Balkan Peninsula, and in the Mediterranean islands (Elba, Baleares, Malta, Corsica, Sardinia, Cyprus, Crete). Indeed, seroprevalence studies in both human and non-human vertebrates have revealed significant infection rates, with high prevalence reported in Italy (19.8%), Türkiye (17.8%), Greece (21%) and North Africa (22%−41%) indicating the widespread presence of TOSV across the Mediterranean basin [16,17,18].

Cases of imported TOSV infection, acquired by travelers in TOSV endemic areas, were diagnosed upon their return home in the US, in the UK, in Switzerland, in Australia, in the Czech Republic and in Northern Europe countries [16]. These cases corresponded to severe infections, mostly with neurological manifestations, that justified hospitalization, allowing for specific investigations leading to TOSV diagnosis. Moreover, in a recent retrospective study in Germany, Dersch et al. reported cases of TOSV neuroinvasive disease in patients without recent travel to endemic regions, indicating a potentially broader geographical distribution of the virus than previously recognized [19].

In the period 2023–2025, national arboviral surveillance data from the Italian National Institute of Health (Istituto Superiore di Sanità, ISS) documented ongoing transmission of TOSV across multiple regions of Italy, registering a total of 376 neuroinvasive cases, the majority of which were autochthonous. Among Italian regions, Emilia-Romagna and Tuscany emerged as principal contributors to the national burden of confirmed cases, with increasing cases reported also in other regions like Lombardia, Piemonte, Veneto, Liguria, Abruzzo and Lazio [20].

The accumulating number of cases and the rare inclusion of TOSV in the diagnostic algorithm of nervous system infections (CNS) confirm its role as a persistent arboviral threat in endemic areas and demonstrate the importance of improving TOSV infection surveillance.

Therefore, to correctly investigate and eventually mitigate the impact of this virus on human health is crucial performing genetic characterization and phylogenetic studies, which are essential to understand viral evolution, host adaptation, and potential markers of pathogenesis.

It has been documented that the use of metagenomic Next-Generation Sequencing (mNGS) allowed simultaneous detection of viral sequences and comprehensive genomic analysis, overcoming the limitations of traditional PCR or culture-based methods and providing a high-resolution view of viral genetic variation [13]. Recently, Brandolini et al. investigated strategies to develop a standardized and efficient workflow for whole-genome sequencing of TOSV, using a commercial library preparation kit on biological samples and sandfly pools to facilitate an efficient method for comprehensive genomic characterization of TOSV [21]. Similarly, they suggest the importance of understanding the TOSV genetic variability combining genomic data with epidemiological and clinical information, leading to a better understanding of how TOSV spreads and evolves over time and improving surveillance and public health responses.

In this work, we provide a comprehensive genomic characterization of TOSV strains detected in Italy over a three-year period, combining viral isolation, target enrichment, and metagenomic Next-Generation Sequencing.

A total of thirty-two TOSV isolates, from human samples, and two, from sandfly homogenates, originating from regions of central–northern Italy, have been selected for this study.

All the isolates were sequenced using an enrichment panel protocol (IlluminaTM) followed by mNGS sequencing, achieving a mean genome coverage of approximately 90% and a mean sequencing depth of 250X. Then, the phylogenetic analysis of the S and M segments demonstrated that all isolates clustered within or near Lineage A (Figure 1 and Figure 2), with no evidence of Lineage B circulation in the sampled regions.

The isolates formed well supported subclades (bootstrap values ≥ 90), with short terminal branches and limited intra cluster diversity, suggesting a recent common ancestry and ongoing local transmission rather than repeated introductions. The strong bootstrap support observed in most internal nodes reinforces the robustness of the phylogenetic inference. These findings are consistent with previous reports of Lineage A predominance in Italy and reflect a relatively stable epidemiological scenario over recent years [3,13].

Regional diversity analysis was carried out to compare intra Tuscany genetic distances with inter regional divergence, testing for geographic distribution of TOSV populations.

The study revealed that Tuscany isolates (*n* = 10; Siena) formed a distinct subclade (bootstrap 95%) with significant lower intra group divergence [S: d = 0.030 (±0.004); M: d = 0.039 (±0.003)] compared to inter regional distances [S: d = 0.043–0.050; M: d = 0.037–0.049; *p* < 0.05, unpaired *t*-test]. Evolutionary analyses revealed that both S (ω = 0.02) and M (ω = 0.48) segments are subjected to negative selection, constraining non-synonymous mutations especially in the S segment nucleoprotein critical for replication and ribonucleoprotein assembly [4].

The mutation rate per base appeared similar in the S (3.84 × 10^−2^ per site) and M (3.27 × 10^−2^ per site) segments (*p* = 0.12). The observed non-synonymous mutations, although limited in number (seven in S, nine in M), may contribute to viral adaptation, antigenicity, or vector competence, and warrant further functional characterization.

Our results highlight the utility of mNGS for genomic surveillance of TOSV, enabling high-resolution detection and characterization of viral sequences and facilitating the recognition or emergence of variants. The integration of metagenomic sequencing with classical epidemiological and clinical data can facilitate the identification of emerging variants, inform public health strategies, and guide vector control efforts. Extending previous research by Marsili et al. [11], who described the characterization of three TOSV isolates collected from Tuscany region, we performed a broader analysis including a larger dataset and gaining a deeper insight into the circulation of TOSV in central/northern Italy regions.

Some limitations, however, should be acknowledged. Indeed, the whole-genome sequencing was not performed directly on all the original samples due to the limited volume of material available and the absence of informed consent from all patients. Therefore, virus isolation in Vero E6 cell culture was required prior to sequencing. Although propagation in cell culture may determine viral adaptation and genetic variability, sequencing was performed after the first cell passage only, thereby minimizing the culture-induced variations. In addition, previous studies on arboviruses, including Orthobunyaviruses and Phleboviruses, have shown that a small number of passages in cell culture generally do not substantially alter the consensus genome [22,23,24,25,26].

These findings are in line with our preliminary mNGS analysis performed on a limited set of primary samples and the corresponding isolates after a single passage on Vero E6 cells. Indeed, no additional genomic mutations were detected.

Therefore, although the possibility that in vitro propagation may have contributed to some of the observed mutations cannot be completely excluded, the overall impact of cell culture on the genomic features reported here is likely negligible. Moreover, the relatively small sample size and the limited geographic coverage may underestimate the overall genetic diversity and the potential circulation of other lineages, such as Lineage B or the recently described Lineage C [4,10].

The genome sequence of circulating TOSV strains in Veneto was made possible through the genomic analyses on sandfly homogenates since human samples were not available. In addition, the incomplete coverage of the L segment, which limited the complete whole-genome sequencing, prevented its inclusion in phylogenetic analyses.

Future studies should aim to expand sampling both geographically and temporally, include additional vector species, and explore the functional consequences of observed mutations through in vitro and in vivo experiments.

In conclusion, this study provides novel genomic insights into TOSV circulation, genetic diversity and evolutionary dynamics across central/northern Italy (2022–2025). The predominance of Lineage A limited intra-lineage variability, and evidence of negative selection across genomic segments suggests a relatively stable viral population. The application of mNGS enhances our understanding of TOSV epidemiology and highlights its potential for guiding surveillance and control strategies in endemic regions.

## Figures and Tables

**Table 1:** Principal mutations detected in the protein sequence of the sequenced samples.

Segment	Mutation
Segment S	180 K > M; 217 T > I; 254 C > S; 280 A > V; 274 A > V; 284 P > L; 302 R > T.
Segment M	268 D > S; 272 G > R; 426 I > T; 470 G > S; 507 P > S; 793 T > A; 995 L > S; 1063 R > L; 1112 F > S.

**Table 2:** Genetic divergence among TOSV isolates by region pairwise mean evolutionary distances (Jukes–Cantor model) on S and M segments, calculated using Geneious v2025.0.2. N indicates the number of isolates (n_1_ × n_2_ for intergroup comparisons); *p*-values from unpaired *t*-test (Tuscany vs. other regions). “Extra Tuscany (combined)” represents intra group variability across Emilia-Romagna (*n* = 22), Lombardia (*n* = 2), and Veneto (*n* = 2; total *n* = 24).

Comparison	N (n_1_ × n_2_)	S Segment (d)	M Segment (d)	*p*-Value (*t*-Test)
Intra Tuscany	10	0.030	0.039	-
Tuscany vs. Emilia-Romagna	10 × 22	0.050	0.049	0.025
Tuscany vs. Lombardia	10 × 2	0.043	0.042	-
Tuscany vs. Veneto	10 × 2	0.045	0.037	0.023
Extra Tuscany regions (combined)	24	0.044	0.061	-

## Data Availability

Datasets analysed or generated during the study can be provided, upon reasonable request, by contacting the corresponding author (mariagrazia.cusi@unisi.it).

## Supplementary Materials

The following supporting information can be downloaded at: https://www.mdpi.com/article/10.3390/pathogens15030338/s1, Table S1: Nucleotides mutation founded in S and M segments. Figure S1: Segment M original high-fidelity phylogenetic tree. It has been reconstructed using Maximum likelihood method. The percentage of replicate trees in which the associated taxa clustered together (1000 replicates) is shown next to the branches. Branch lengths are proportional to evolutionary distance; the scale bar represents substitutions per site. Orange icons indicate samples collected from Emilia Romagna region, red icons samples from Tuscany region, violet icons samples from Veneto and green icons samples from Lombardia. Figure S2: Segment S original high-fidelity phylogenetic tree. It has been reconstructed using Maximum likelihood method. The percentage of replicate trees in which the associated taxa clustered together (1000 replicates) is shown next to the branches. Branch lengths are proportional to evolutionary distance; the scale bar represents substitutions per site. Orange icons indicate samples collected from Emilia Romagna region, red icons samples from Tuscany region, violet icons samples from Veneto and green icons samples from Lombardia.
