# Supplementary material for: Molecular Epidemiology of Toscana Virus in Northern and Central Italy Using Metagenomic Next-Generation Sequencing
Source: Pathogens. 2026 Mar 21;15(3):338. doi: 10.3390/pathogens15030338 (PMC13029180; doi:10.3390/pathogens15030338)
Supplement: Supplementary file 1 [file pathogens-15-00338-s001.zip › Supplementary Figures S1,S2.pdf]

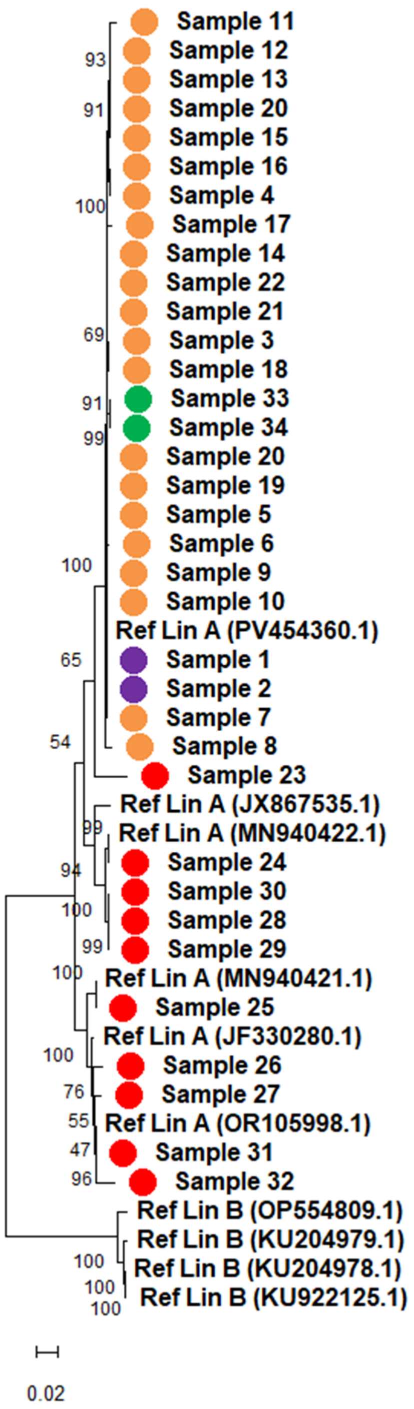

Figure 1. **Original high-fidelity phylogenetic tree of M segment**. It has been reconstructed using Maximum likelihood method. The percentage of replicate trees in which the associated taxa clustered together (1000 replicates) is shown next to the branches. Branch lengths are proportional to evolutionary distance; the scale bar represents substitutions per site. Orange icons indicate samples collected from Emilia Romagna region, red icons samples from Tuscany region, violet icons samples from Veneto and green icons samples from Lombardia.

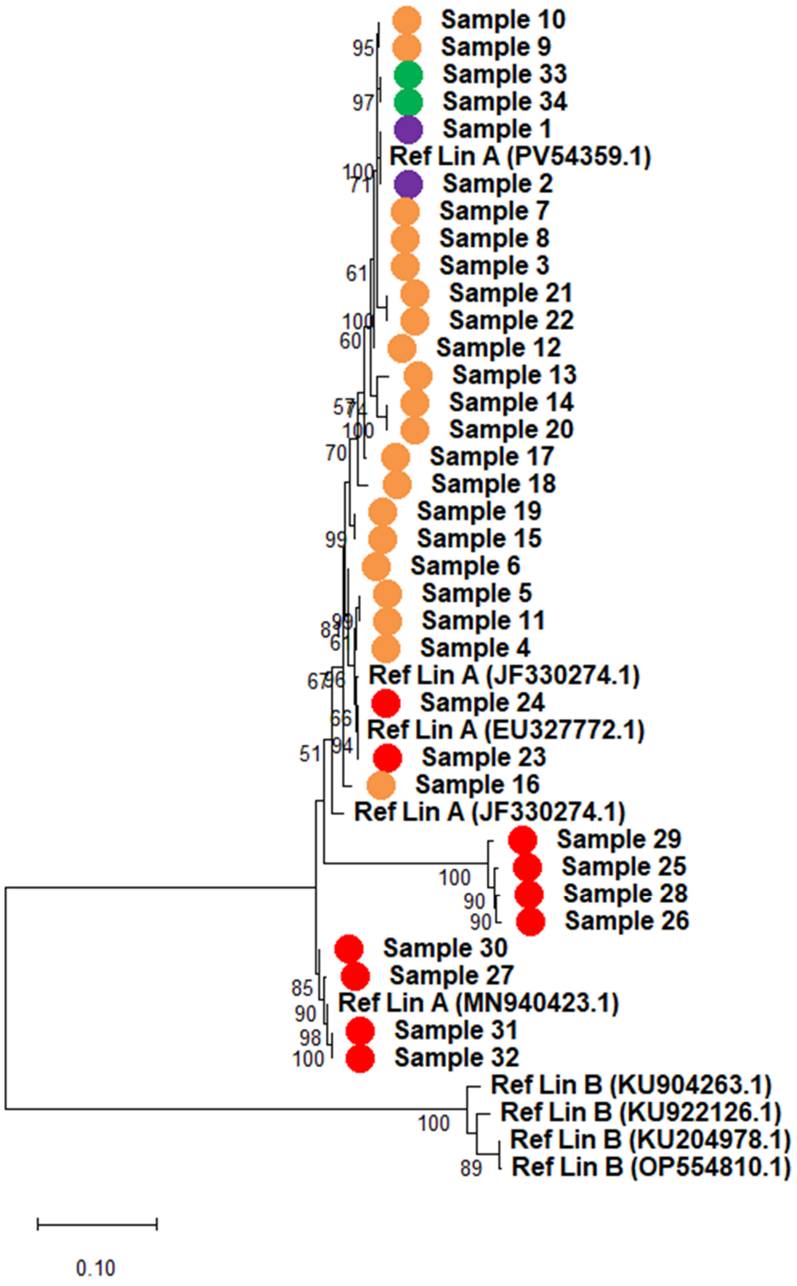

Figure 2. Original high-fidelity phylogenetic tree of S segment. It has been reconstructed using Maximum likelihood method. The percentage of replicate trees in which the associated taxa clustered together (1000 replicates) is shown next to the branches. Branch lengths are proportional to evolutionary distance; the scale bar represents substitutions per site. Orange icons indicate samples collected from Emilia Romagna region, red icons samples from Tuscany region, violet icons samples from Veneto and green icons samples from Lombardia.
