# Supplementary material for: Molecular Epidemiology of Toscana Virus in Northern and Central Italy Using Metagenomic Next-Generation Sequencing
Source: Pathogens. 2026 Mar 21;15(3):338. doi: 10.3390/pathogens15030338 (PMC13029180; doi:10.3390/pathogens15030338)
Supplement: Supplementary file 1 [file pathogens-15-00338-s001.zip › Supplementary Table S1.pdf]

**Table S1:** Nucleotides mutation founded in S and M segments.

| **Segment** | **Mutation (c.)** |
|---|---|
| **Segment S** | c.62T>C; c.140A>G; c.146T>C; c.185G>A; c.200C>T; c.242C>T; c.248A>G; c.275G>A; c.323C>T; c.327T>C; c.341C>T; c.356C>T; c.374G>A; c.380C>T; c.449T>C; c.470A>G; c.494C>T; c.509G>A; c.530G>A; c.536A>G; c.542A>G; c.656T>C; c.677A>G; c.686C>T; c.695T>C; c.701G>A; c.731T>C; c.758A>G; c.824A>G; c.830G>A; c.846A>G; c.905C>T; c.924A>G; c.948G>A; c.1004G>A; c.1016A>T; c.1025A>G; c.1046G>A; c.1051T>A; c.1089C>T; c.1091T>C; c.1106A>G; c.1133C>T; c.1139G>A; c.1190C>T; c.1205T>C; c.1232A>G, c.1286T>C; c.1385C>T; c. 1405A>G, c.1421G>A; c.1448A>G; c.1497C>T, c.1532C>T; c.1541A>G; c.1546G>T; c.1580A>G; c.1592C>T; c.1613A>C; c.1625T>C; c.1657T>C; c.1657T>C; c.1664G>A; c.1673A>G; c.1673A>G; c.1673A>G; c.1676G>A; c.1704T>C; c.1756A>G, c.1766G>A; c.1786A>G; c.1830A>G; c.1833A>G. |
| **Segment M** | c. 224A>G; c.230G>A; c.377C>T; c.395G>A; c.404A>G; c.422T>C; c.426A>G; c.443A>G; c.461A>G; c.488G>A; c.498A>G; c.522T>C; c.515C>T; c.523C>G; c.543C>T; c.575A>T; c.591A>T; c.611G>A; c.617G>A; c.625G>A; c.644A>G; c.646G>A; c.669A>G; c.671T>C; c.680A>G; c.683A>G; c.740A>G; c.779C>T, c.802A>T; c.811T>G; c.830T>C; c.850G>A; c.881G>A; c.911G>A; c.980T>C; c.1029A>G; c.1053T>C; c.1109A>G; c.1121A>G, c.1181T>C, c.1187A>G; c.1199C>T; c.1208T>C; c.1234A>G; c.1307T>C; c.1352T>C; c.1393G>A; c.1394A>G; c.1397C>T; c.1417G>A; c.1452T>C; c.1457C>T; c.1466G>A; c.1502A>G; c.1514C>T; c.1526A>G; c.1559A>G; c.1562A>G; c.1613T>C; c.1625A>G; c.1629T>C; c.1664T>C; c.1745T>C; c.1748T>C; c.1755T>C; c.1757A>T; c.1776A>G: c.1796C>T; c.1803G>A; c.1854C>T; c.1860A>G; c.1862G>T; c.1928C>T; c.1943C>T; c.2006G>A; c.2019C>T; c.2021A>G; c.2132C>T; c.2160G>A; c.2174T>C; c.2205C>T, c.2219T>C; c.2241T>C; c.2264G>A; c.2284A>G; c.2288A>G; c.2303T>C; c.2340A>G; c.2362A>G; c.2362A>G; c.2334A>G; c.2399T>C; c.2408T>A; c.2477T>C; c.2492G>A; c.2496A>G; |

| | |
|---|---|
| | c.2546C>T; c.2556G>A; c.2612G>A; c.2666T>C; c.2672C>T; c.2674A>G; c.2678A>G; c.2681G>A; c.2687C>T; c.2702A>C; c.2774T>C; c.2792A>G; c.2876A>G; c.2894A>G; c.2924T>C; c.2966T>C; c.2996C>T; c.3026C>T; c.3098C>G; c.3167C>T; c.3266G>A; c.3302T>C; c.3314C>T; c.3365T>C; c.3404T>C; c.3407G>A; c.3491T>C; c.3518T>C; c.3521G>A; c.3590A>C; c.3665T>C; c.3677G>A; c.3679G>A; c.3788T>C; c.3821 G>A; c.3836T>C; c.3846G>A; c.3875C>T; c.3884G>A; c.3933T>C; c.3988C>T; c.4021A>G. |
